# Unregulated miR-96 Induces Cell Proliferation in Human Breast Cancer by Downregulating Transcriptional Factor FOXO3a

**DOI:** 10.1371/journal.pone.0015797

**Published:** 2010-12-23

**Authors:** Huanxin Lin, Ting Dai, Huaping Xiong, Xiaohui Zhao, Xiuting Chen, Chunping Yu, Jun Li, Xi Wang, Libing Song

**Affiliations:** 1 State Key Laboratory of Oncology in Southern China, Department of Experimental Research, Cancer Center, Sun Yat-sen University, Guangzhou, China; 2 Department of Radiotherapy, Cancer Center, Sun Yat-sen University, Guangzhou, China; 3 Department of Biochemistry, Zhongshan School of Medicine, Sun Yat-sen University, Guangzhou, China; 4 Department of Microbiology, Zhongshan School of Medicine, Sun Yat-sen University, Guangzhou, China; 5 Department of Breast Surgery, Cancer Center, Sun Yat-sen University, Guangzhou, China; IDI-IRCCS, Italy

## Abstract

FOXO transcription factors are key tumor suppressors in mammalian cells. Until now, suppression of FOXOs in cancer cells was thought to be mainly due to activation of multiple onco-kinases by a phosphorylation-ubiquitylation-mediated cascade. Therefore, it was speculated that inhibition of FOXO proteins would naturally occur through a multiple step post-translational process. However, whether cancer cells may downregulate FOXO protein via an alternative regulatory mechanism is unclear. In the current study, we report that expression of miR-96 was markedly upregulated in breast cancer cells and breast cancer tissues compared with normal breast epithelial cells (NBEC) and normal breast tissues. Ectopic expression of miR-96 induced the proliferation and anchorage-independent growth of breast cancer cells, while inhibition of miR-96 reduced this effect. Furthermore, upregulation of miR-96 in breast cancer cells resulted in modulation of their entry into the G1/S transitional phase, which was caused by downregulation of cyclin-dependent kinase (CDK) inhibitors, p27^Kip1^ and p21^Cip1^, and upregulation of the cell-cycle regulator cyclin D1. Moreover, we demonstrated that miR-96 downregulated FOXO3a expression by directly targeting the FOXO3a 3′-untranslated region. Taken together, our results suggest that miR-96 may play an important role in promoting proliferation of human breast cancer cells and present a novel mechanism of miRNA-mediated direct suppression of FOXO3a expression in cancer cells.

## Introduction

The FOXO subfamily of Forkhead transcription factors, including FoxO1 (FKHR), FoxO3a (FKHRL1), FoxO4 (AFX) and FoxO6 contains evolutionarily conserved transcriptional activators that are characterized by a highly conserved forkhead domain with a DNA-binding motif [1]. FOXO proteins play a pivotal role in biological processes, such as apoptosis, cell cycle control, differentiation, stress response, DNA damage repair and glucose metabolism [2]. Activation of each member of the FOXO subfamily in cells can upregulate cell-cycle inhibitors p21^Cip1^ and p27^Kip1^ and downregulate the cell cycle regulator cyclin D1/2 (cell-cycle related genes), consequently leading to G1/S arrest of cells [3]–[5]. It has been also reported that upregulation of FOXO proteins can induce apoptosis through regulation of multiple pro-apoptotic proteins, including Bim, Puma, Fas ligand and TRAIL [6]–[9]. Meanwhile, FOXO proteins have been associated with DNA damage repair via upregulation of GADD45a or interaction with ATM to promote DNA repair via downstream mediators [10]–[12]. Therefore, FOXO transcription factors are considered key tumor suppressors. Indeed, downregulation of FOXO1 in chicken embryo fibroblasts or inhibition of transcriptional activity of FOXO3a protein in human breast cancer cells can promote cell transformation and tumor progression [13]–[14]. Broad somatic deletion of all FOXOs in mice were shown to promote a progressive cancer-prone condition characterized by thymic lymphomas and hemangiomas, and stable introduction of a dominant-negative FOXO moiety into Eμ-myc transgenic hematopoietic stem cells could accelerate lymphoma development in recipient mice [15]–[16]. These observations demonstrate that the mammalian FOXOs are *bona fide* tumor suppressors.

The inhibition of cell proliferation and survival by FOXO transcription factors is often abrogated due to high level activation of multiple onco-kinases in cancer cells, such as Akt, SGK1 (serum-and glucocorticoid-inducible kinase 1) and IκB kinase (IKK)-β [17], [18], [14]. Phosphorylation of FOXO transcriptional factors can result in their release from the DNA and translocation from the nucleus to cytoplasm through interaction with 14-3-3 chaperone proteins [19]. Although activation of the abovementioned onco-kinases can contribute to persistent phosphorylation and degradation of FOXO proteins, we wondered why cancer cells would downregulate FOXO proteins via multiple steps (such as phosphrylation, nuclear/cytoplasmic translocation, and ubiquitin-mediated degradation) rather than halt synthesis at the translational step as it is energy-consuming for the cell to continually re-synthesize and re-degrade these proteins. Thus, we hypothesized that there may be an alternative regulatory mechanism of FOXO protein expression in cancer.

MicroRNAs (miRNAs), a class of small non-coding RNAs, regulate gene expression by inhibition of translation or facilitation of mRNA degradation that result in repression of target genes by binding to the 3′-UTR of a target mRNA molecule [20]–[21]. Numerous studies have reported that miRNAs are involved in the development and progression of various types of human cancers and proposed as potential novel targets for anti-cancer therapies [22]–[24].

In the current study, the expression of miR-96 in breast cancer cells was compared to that in normal tissue, and the effect of its overexpression on the proliferation of tumor cells was investigated. We determined that miR-96 likely promotes breast cancer proliferation by directly targeting the 3′untranslated region (3′-UTR) of the FOXO3a mRNA, consequently reducing the expression of cyclin-dependent kinase (CDK) inhibitors, p27^Kip1^ and p21^Cip1^, and upregulating the cell-cycle regulator cyclin D1. Our results suggest that miR-96 may play an important role in the development and progression of breast cancer.

## Materials and Methods

### Ethics Statement

#### Normal breast samples

Normal breast samples was collected from the mammoplasty material of a 30-year-old woman and approved by the Sun Yat-sen University and First Affiliated Hospital Institutional Board. Samples was collected and analyzed with written informed consent.

#### Breast cancer tissue specimens

This study was conducted on a total of 23 breast cancer samples which were histopathologically and clinically diagnosed at the Sun Yat-sen University Cancer Center from 2009 to 2010. Clinical and clinicopathological classification and staging were determined according to the American Joint Committee on Cancer (AJCC) criteria [25]. The stage and grade of primary breast tumors were as follows: 3 cases at stage I of disease, 12 at stage II, 7 at stage III, and 1 at stage IV. Of these breast cancers samples, 14 were ERα-positive, 16 were PR-positive, and 18 were HER2-positive. For the use of clinical materials for research purposes, prior patients' consents and approval was obtained from the Sun Yat-sen University and Cancer Center Institutional Board. All samples were collected and analyzed with prior written informed consent from the patients.

### Cell Culture

Primary NBEC, form mammoplasty material, were cultured in the Keratinocyte serum-free medium (Invitrogen, Carlsbad, CA) supplemented with epithelial growth factor, bovine pituitary extract and antibiotics (120 µg/ml streptomycin and 120 µg/ml penicillin). Breast cancer cell lines, including MCF-7, ZR-75-30, BT549, Bcap37, MDA-MB435, SKBR3, MDA-MB453 and T47D, were maintained in DMEM medium (Invitrogen, Carlsbad, CA) supplemented with 10% fetal bovine serum (HyClone, Logan, UT) and 1% penicillin/streptomycin (Invitrogen, Carlsbad, CA, USA).

### Plasmid and transfection

The full-length sequence of FOXO3a-3′UTR is 4977 base pairs (bp) long and contains two conserved miR-96 binding sites (REs), including RE#1 from 76 bp to 80 bp, and RE#2 from 916 bp to 922 bp; one reported miR-155 binding site from 1021 bp to 1027 bp [26]; two reported miR-182 binding sites (REs), including RE#1 from 73 bp to 79 bp, and RE#2 from 915 bp to 921 bp [27]. The region of human FOXO3a 3′UTR, from 47 to 939, generated by PCR amplification from NBEC, were cloned into the Sac I/Xma I sites of the pGL3-basic luciferase reporter plasmid (Promega, Madison, WI) and pGFP-C3 (Clontech, Mountain View, CA). The primers selected are as the following: FOXO3a-3′UTR-wt-up: 5′- AAGACCTACAGAGAAAACCCTTTGCCAAATCTGCTCTC AGC-3′; FOXO3a-3′UTR-wt-dn: 5′-CTAAACCCTTTAGTGACATTTGGCAATGAGTGG CCCGG-3′; FOXO3a-3′UTR-mu-up: 5′-AAGACCTACAGAGAAAACCCTTTGCCACC TCTGCTCTCAGC-3′; FOXO3a-3′UTR-mu-dn: 5′-CTAAACCCTTTAGTGACATGGGGC AATGAGTGGCCCGGG-3′; p3x IRS-MLP-luc plasmid was constructed as previously described [28]. The miR-96 mimics, negative control, and anti-miR-96 inhibitor were purchased from RiboBio (RiboBio Co.Ltd, Guangzhou, Guangdong). Transfection of microRNA or microRNA inhibitor was performed using the Lipofectamine 2000 reagent (Invitrogen) according to the manufacturer's instruction.

### Western blotting

The western blot analysis was performed according to standard methods as previously described [29], using anti-FOXO3a, anti-p21, anti-p27, anti-cyclinD1, anti-Ki-67 antibodies (Cell Signaling, Danvers, MA), and anti-Rb, anti- phosphorylated Rb antibodies (Abcam, Cambridge, MA). The membranes were stripped and re-blotted with an anti-α-tubulin monoclonal antibody (Sigma, Saint Louis, MO) as a loading control.

### RNA Extraction and Real-Time Quantitative PCR

Total miRNA from cultured cells and fresh surgical breast cancer tissues was extracted using the mirVana miRNA Isolation Kit (Ambion, Austin, TX, USA) according to the manufacturer's instructions. cDNA was synthesized from 5 ng of total RNA using the Taqman miRNA reverse transcription kit (Applied Biosystems, Foster City, CA), and the expression levels of miR-96 were quantified using miRNA-specific TaqMan MiRNA Assay Kit (Applied Biosystems). Real-time PCR was performed using the Applied Biosystems 7500 Sequence Detection system. The primers selected are as the following: p21^Cip1^, forward, 5′-CGATGCCAACCTCCTCAACGA -3′, and reverse, 5′-TCGCAGACCT CCAGCATCCA-3′; p27^Kip1^, forward, 5′-TGCAACCGA CGATTCTTCTACTCAA-3′, and reverse, 5′-CAAGCAGTGATGTATCTGATAAACAAGG A-3′; cyclin D1, forward, 5′- AAC TACCTGGACCGCTTCCT -3′, and reverse, 5′-CCACTT GAGCTTGTTCACCA-3′). Expression data were normalized to the geometric mean of housekeeping gene *GAPDH* to control the variability in expression levels (forward primer, 5′-GACTCATGACCACAGTCCATGC-3′; reverse primer, 3′-AGAGGCAGGGATGATG TTCTG -5′) and calculated as 2^-[(Ct of *p21, p27, or cyclin D1*) – (Ct of *GAPDH*)]^, where C_t_ represents the threshold cycle for each transcript. The expression of miRNA was defined based on the threshold cycle (Ct), and relative expression levels were calculated as 2^-[(Ct of miR-96) – (Ct of U6)]^ after normalization with reference to expression of U6 small nuclear RNA.

### 3-(4, 5-Dimethyl-2-thiazolyl)-2, 5-diphenyl-2H-tetrazolium bromide (MTT) assay

Cells, seeded on 96-well plates, were stained at indicated time point with 100 µl sterile MTT dye (0.5 mg/ml, Sigma) for 4 h at 37°C, followed by removal of the culture medium and addition of 150 µl of dimethyl sulphoxide (DMSO) (Sigma, St. Louis, MO, USA). The absorbance was measured at 570 nm, with 655 nm as the reference wavelength. All experiments were performed in triplicates.

### Anchorage-independent growth ability assay

Five hundred cells were trypsinized and suspended in 2 ml complete medium plus 0.3% agar (Sigma, St Louis, MO). The agar–cell mixture was plated on top of a bottom layer with 1% complete medium agar mixture. After 10 days, viable colonies that contained more than 50 cells or were larger than 0.1 mm were counted. Colony size was measured with an ocular micrometer and colonies greater than 0.1 mm in diameter were counted. The experiment was performed for three independently times for each cell line.

### Colony formation assays

Cells were plated on 6-well (0.5×10^3^ cells per plate) and cultured for 10 days. The colonies were stained with 1.0% crystal violet for 30s after fixation with 10% formaldehyde for 5 min.

### Bromodeoxyuridine labeling and immunofluorescence

Cells grown on coverslips (Fisher, Pittsburgh, PA) were incubated with bromodeoxyuridine (BrdUrd) for 1 h and stained with anti-BrdUrd antibody (Upstate, Temecula, CA) according to the manufacturer's instruction. Gray level images were acquired under a laser scanning microscope (Axioskop 2 plus, Carl Zeiss Co. Ltd., Jena, Germany).

### Luciferase assays

Cells (3.5×10^4^) were seeded in triplicates in 24-well plates and allowed to settle for 24 h. One hundred nanogram of p3x IRS-MLP -luciferase plasmid, or pGL3-FOXO3a-3′UTR(wt/mu), or the control-luciferase plasmid, plus 1 ng of pRL-TK renilla plasmid (Promega, Madison, WI), were transfected into breast cancers using the Lipofectamine 2000 reagent (Invitrogen Co., Carlsbad, CA) according to the manufacturer's recommendation. Luciferase and renilla signals were measured 48 h after transfection using the Dual Luciferase Reporter Assay Kit (Promega, Madison, WI) according to a protocol provided by the manufacturer. Three independent experiments were performed and the data are presented as the mean ± SD.

### Flow cytometry analysis

All cells in a culture dish were harvested by trypsinization, washed in ice-cold PBS, and fixed in 80% ice-cold ethanol in PBS. Before staining, the cells were spun down in a cooled centrifuge and resuspended in the cold. Bovine pancreatic RNAase (Sigma-Aldrich) was added at a final concentration of 2 µg/ml, and cells were incubated at 37°C for 30 min, followed by incubation in 20 µg/ml of propidium iodide (Sigma-Aldrich) for 20 min at room temperature. 50,000 cells were analyzed on a flow cytometer (FACSCalibur; BD Biosciences).

### Statistical analysis

The Student's t -test was used to evaluate the significant difference of two groups of data in all the pertinent experiments. A *P* value <0.05 (using a two-tailed paired t test) was thought to be significantly different for two groups of data.

## Results

### miR-96 is overexpressed in breast cancer cell lines and breast cancer tissues

**Figure 1:**
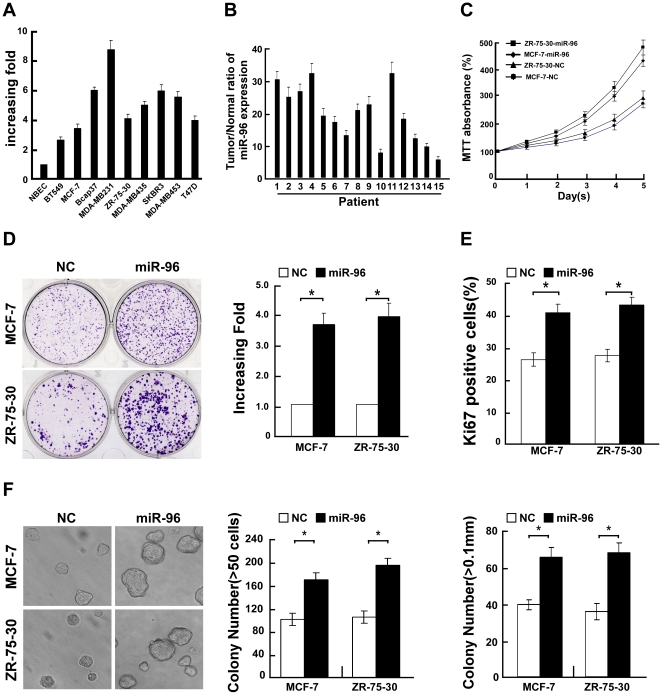
Upregulation of miR-96 promoted proliferation of breast cancer cells. **A**, Real-time PCR analysis of miR-96 expression in normal breast epithelial cells (NBECs) and breast cancer cell lines, including BT549, ZR-75-30, Bcap37, MDA-MB231, MDA-MB435, MCF-7, SKBR3, MDA-MB453 and T47D. **B**, The expression of miR-96 was examined in 15 paired breast tumor tissues (tumor) and their adjacent normal tissues (normal). The average miR-96 expression was normalized by U6 expression. Each bar represents the mean of three independent experiments. **C**, Effects of miR-96 overexpression on the growth of breast cancer cells MCF-7 and ZR-75-30. MTT assays revealed that miR-96-transfected cells proliferated more rapidly than the vector control cells. **D,** Representative micrographs (left) and quantification (right) of crystal violet stained cell colonies. **E,** Quantification of Ki-67 positive cells in MCF-7 and ZR-75-30 cells transfected with miR-96 mimic and negative control (NC). **F,** Upregulation of miR-96 promoted breast cancer cell tumorigenicity as determined by anchorage-independent growth assay. Representative micrographs (left) and quantification of colonies that contained more than 50 cells (middle) or were larger than 0.1 mm (right) were scored. Each bar represents the mean of three independent experiments. * *P*<0.05.

Real-time PCR analyses revealed that miR-96 was significantly overexpressed in all nine examined breast cancer cell lines, including BT549, ZR-75-30, Bcap37, MDA-MB231, MDA-MB435, MCF-7, SKBR3, MDA-MB453 and T47D, as compared with that in normal breast epithelial cells (NBEC) (Figure 1A). Furthermore, comparative analysis revealed that miR-96 was differentially overexpressed in 15 examined samples paired with adjacent non-cancerous tissues from the same patient (Figure 1B), indicating that miR-96 is upregulated in breast cancer.

### Upregulation of miR-96 enhances proliferation and tumorigenicity of breast cancer cells

To investigate the biological role of miR-96 expression in the development and progression of breast cancer, we next transfected breast cancer cells MCF-7 and ZR-75-30 with hsa-miR-96 mimic oligonucleotides and examined its effect on cellular proliferation. By using MTT and colony formation assays, we observed that overexpression of miR-96 dramatically increased the growth rate of both breast cancer cells as compared with that of negative control (NC) transfected cells (Figure 1C and 1D). Furthermore, the expression of Ki-67, a well-known proliferative marker, was dramatically increased in miR-96 overexpressing-MCF-7 and -ZR-75-30 breast cancer cells compared with that in NC transfected cells (Figure 1E), suggesting that upregulation of miR-96 specifically promotes the proliferation of breast cancer cells. Moreover, ectopically expressing miR-96 in MCF-7 and ZR-75-30 breast cancer cells significantly enhanced their anchorage-independent growth ability, as indicated by the increase in colony numbers and sizes (Figure 1F), suggesting that upregulation of miR-96 could augment the tumorigenicity of breast cancer cells *in vitro*.

### Overexpression of miR-96 regulates G1/S phase transition of breast cancer cells

**Figure 2:**
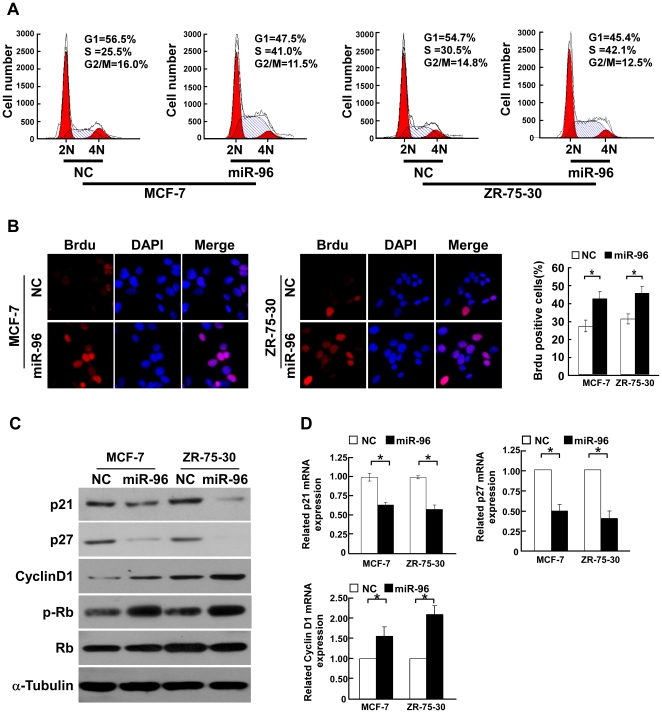
miR-96 induced proliferation through increasing the proportion of S phase cells. **A**, Flow cytometric analysis of indicated breast cancer cells transfected with miR-96 mimic or NC. **B,** Representative micrographs (left) and quantification of BrdU incorporating-cells after transfection with miR-96 or NC. **C,** Western blotting analysis of expression of p21^Cip1^, p27^Kip1^, cyclin D1, phosphorylated Rb (p-Rb) and total Rb protein in indicated cells. α-tubulin was used as a loading control. **D,** Real-time PCR analysis of expression of p21^Cip1^, p27^Kip1^, cyclin D1 in indicated cells. *GAPDH* was used as a loading control. Error bars represent mean ± SD from three independent experiments. * *P*<0.05.

To further explore the ability of miR-96 to promote cell proliferation, we analyzed miR-96 overexpressing MCF-7 and ZR-75-30 cells by flow cytometry, which showed a significant decrease in the percentage of cells in the G1/G0 peak and an increase in the percentage of cells in the S peak (Figure 2A). Furthermore, a higher percentage of miR-96 transfected MCF-7 cells (41.5%) and of miR-96 transfected ZR-75-30 cells (44.7%) showed newly synthesized DNA by the BrdUrd incorporation assay compared with MCF-7 control cells (26.2%) and ZR-75-30 control cells (30.5%) (Figure 2B). Collectively, our data suggest that enhancement of breast cancer cell growth by miR-96 may be mediated through regulation of cellular entry into the G1/S transitional phase.

### miR-96 downregulates cell-cycle inhibitors p21^Cip1^ and p27^Kip1^ and upregulates cell cycle regulator cyclin D1

It is well known that CDK inhibitors p21^Cip1^ and p27^Kip1^ and CDK regulator cyclin D1 are critical in the regulation of G1/S transition [3]–[5]. Strikingly, the Western blotting and real-time PCR analyses revealed that p21^Cip1^ and p27^Kip1^ were downregulated and cyclin D1 was upregulated at both protein and mRNA levels in miR-96-overexpressing cells compared with control cells (Figure 2C and 2D). In parallel, the phosphorylation level of Rb, a downstream target protein of CDK, was associated with the change of expression levels of the abovementioned cell-cycle regulators, further supporting the notion that miR-96 plays an important role in the growth of breast cancer cells (Figure 2C).

### Inhibition of miR-96 reduces the growth rate of breast cancer cells

**Figure 3:**
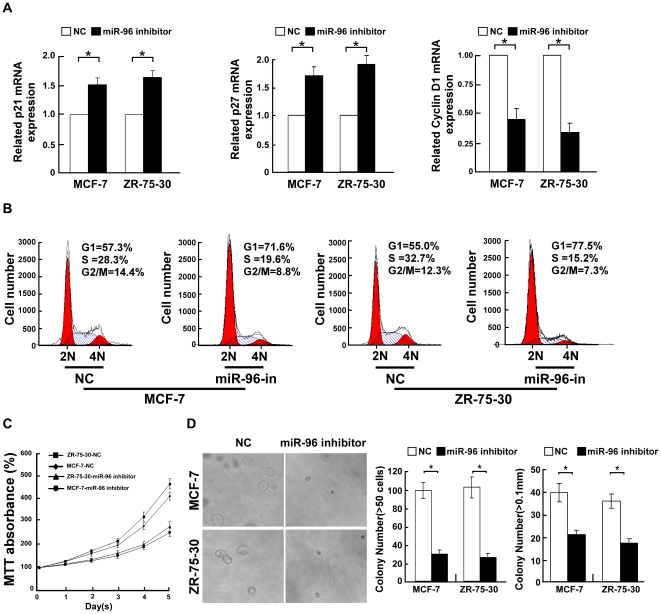
Inhibition of miR-96 suppressed the proliferation of breast cancer cells. **A,** Real-time PCR analysis of expression of p21^Cip1^, p27^Kip1^ and cyclin D1 in indicated cells. *GAPDH* was used as a loading control. Error bars represent mean ± SD from three independent experiments. **B,** Flow cytometric analysis of indicated breast cancer cells transfected with miR-96 inhibitor or NC. **C,** MTT assays revealed that inhibition of miR-96 reduced cell growth. **D,** Representative micrographs (left) and quantification of colonies that contained more than 50 cells (middle) or were larger than 0.1 mm (right) determined by anchorage-independent growth assays. Each bar represents the mean of three independent experiments. * *P*<0.05.

The effect of inhibiting miR-96 on the proliferation of breast cancer cells was further examined. As shown in Figure 3A, suppression of miR-96 could increase the mRNA expression of p21^Cip1^ and p27^Kip1^ while decrease the cyclin D1 transcriptional level. Ectopically expressing the miR-96 inhibitor drastically increased the percentage of cells in the G0/G1 peak but decreased that in the S peak, indicating that inhibition of miR-96 induced G1/S arrest of breast cancer cells (Figure 3B). Moreover, the growth rates in MCF-7 and ZR-75-30 breast cancer cells after transfection with the hsa-miR-96 inhibitor were slower than that of NC transfected cells, as analyzed by an MTT assay (Figure 3C). Inhibition of miR-96 in MCF-7 and ZR-75-30 cells also significantly reduced the anchorage-independent growth of both breast cancer cell lines, as shown by the decrease in the colony numbers and colony sizes (Figure 3D).

### miR-96 directly targets the transcriptional factor FOXO3a in breast cancer cells

**Figure 4:**
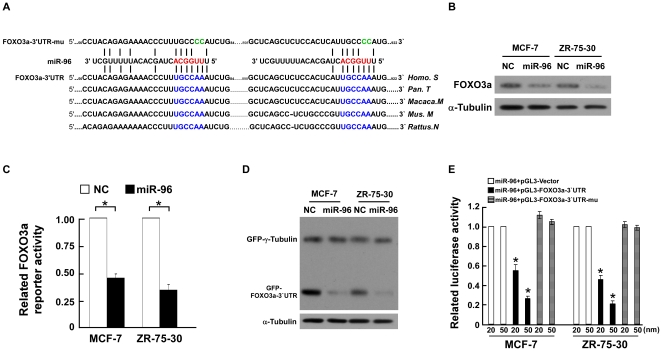
miR-96 downregulated FOXO3a via directly targeting the FOXO3a 3′UTR. **A**, Predicted miR-96 target sequences in 3′-UTR of FOXO3a (FOXO3a) and mutant containing two mutated nucleotides in 3′-UTR of FOXO3a (FOXO3a-mu). **B,** Western blotting analysis of FOXO3a expression in indicated cells. **C,** Relative FOXO3a reporter activities in the indicated cell lines. **D,** Western blotting analysis of GFP expression in indicated cells. **E,** Luciferase assays on MCF-7 or ZR-75-30 breast cancer cells transfected with the pGL3 control reporter, pGL3-FOXO3a-3′UTR reporter, or pGL3-FOXO3a-3′UTR-mu reporter and increasing amounts of miR-96 mimic oligonucleotides (20, 50 nM), as indicated. Error bars represent mean ± SD from three independent experiments. * *P*<0.05.

It has been demonstrated that the transcriptional factor FOXO3a can upregulate the expression of p21^Cip1^ and p27^Kip1^ and downregulate cyclin D1 at the transcriptional level [3]–[5], which prompted us to investigate whether the modulations of p27^ Kip1^, p21^Cip1^ and cyclin D1 levels by miR-96 are caused by the regulation of FOXO3a. Analysis using three publicly available algorithms (TargetScan, Pictar, miRANDA) indicated that FOXO3a is the theoretical target gene of miR-96 (Figure 4A). As predicted, ectopic expression of miR-96 in MCF-7 and ZR-75-30 cells decreased the expression of FOXO3a protein and the FOXO3a transcriptional activity, respectively (Figure 4B and 4C), indicating that upregulation of miR-96 results in the downregulation and decreased transactivity of FOXO3a. Consistent with this result, we found that FOXO3a was downregulated in the breast cancer cells where mir-96 was upregulated (Figure S1 and Figure 1A). Furthermore, we subcloned the FOXO3a 3′-untranslated region (3′-UTR) fragment containing the two miR-96 binding sites (REs) into the pEGFP-C3 and pGL3 dual luciferase reporter vectors. As shown in Figure 4D, ectopic expression of miR-96 in MCF-7 and ZR-75-30 breast cancer cells dramatically inhibited the GFP protein expression, but not the expression of GFP-γ-tubulin that was used as a control for transfection efficiency, suggesting that miR-96 specifically affected the FOXO3a-3′ UTR. Meanwhile, a consistent and dose-dependent reduction of luciferase activity was observed upon miR-96 transfection in both breast cancer cell lines (Figure 4E). Moreover, point mutations in the tentative miR-96-binding seed region in FOXO3a 3′-UTR abrogated the suppressive effect of FOXO3a mediated by miR-96 (Figure 4E). Taken together, our results demonstrate that FOXO3a is a *bona fide* target of miR-96.

**Figure 5:**
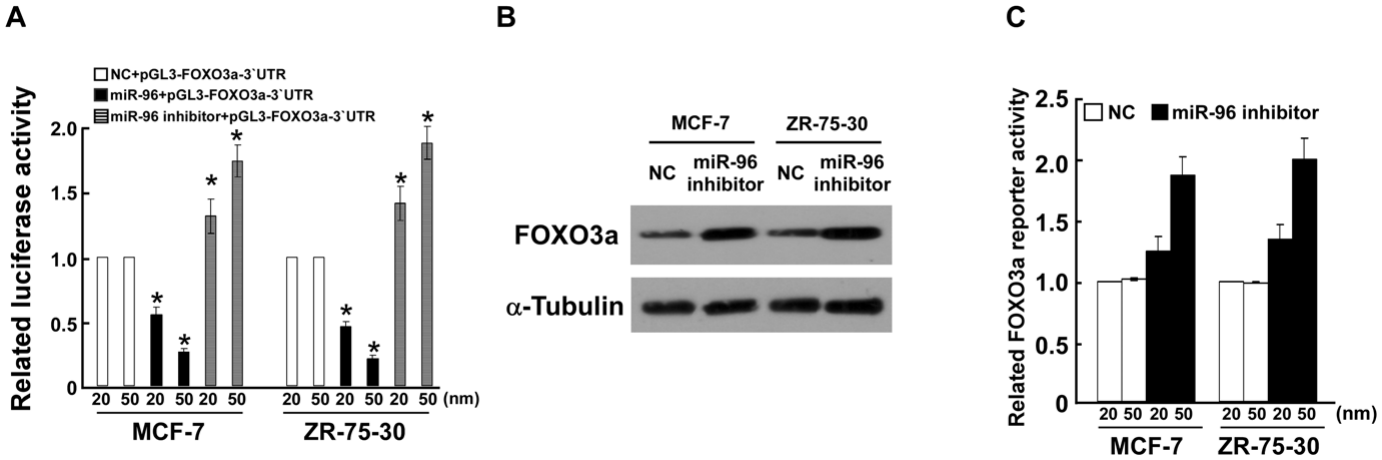
Inhibition of miR-96 activated the FOXO3a pathway. **A,** Luciferase activity assay of indicated cells transfected with the pGL3-FOXO3a-3′UTR reporter with increasing amounts (20, 50 nM) of miR-96 mimic- or miR-96 inhibitor-oligonucleotides. **B,** Western blotting analysis of FOXO3a expression in indicated cells. **C,** Relative FOXO3a reporter activities in the indicated cell lines. Error bars represent mean ± SD from three independent experiments. * *P*<0.05.

As expected, the luciferase levels of pGL3-FOXO3a-3′UTR in both breast cancer cell lines were restored after transfection with the miR-96 inhibitor (Figure 5A). Inhibiting miR-96 also resulted in the upregulation of FOXO3a proteins (Figure 5B). As shown by the luciferase reporter assay, inhibition of miR-96 decreased transactivities of FOXO3a in a dose-dependent manner in both breast cancer cell lines (Figure 5C), indicating that inhibition of miR-96 results in upregulation and increased transactivity of FOXO3a.

### FOXO3a plays an important role in miR-96-induced proliferation of breast cancer cells

**Figure 6:**
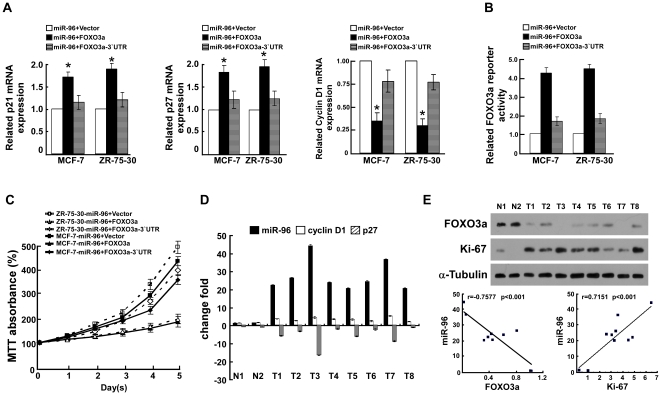
FOXO3a plays an important role in miR-96-induced proliferation. **A,** The expression of p21^Cip1^, p27^Kip1^ and cyclin D1 in indicated cells using real-time PCR analysis. *GAPDH* was used as a loading control. **B,** Relative FOXO3a reporter activity in the indicated cell lines. **C,** The growth rate of breast cancer cells transfected with miR-96 mimic, or mixed miR-96 and FOXO3a, or mixed miR-96 and FOXO3a-3′-UTR. **D,** Real-time PCR analysis of expression of miR-96, p27 and cyclin D1. **E,** Expression of FOXO3a, Ki67 (upper) and correlation (lower) analysis of miR-96, FOXO3a, Ki-67 in two normal human breast tissues and eight freshly prepared human breast cancer tissues. * *P*<0.05.

In the attempt to understand the role of FOXO3a repression in miR-96-induced proliferation, the effects of FOXO3a (without 3′-UTR) and FOXO3a-3′-UTR (with 3′-UTR) were examined in the miR-96-overexpressing cells. As predicted, ectopically expressing FOXO3a significantly abrogated the miR-96 mediated-modulations of cell-cycle regulators, p27^ Kip1^, p21^Cip1^ and cyclin D1, but overexpression of FOXO3a-3′-UTR only partially attenuated the regulation of the abovementioned cell-cycle regulators by miR-96 overexpression (Figure 6A). In particular, the luciferase activity of FOXO3a reporter in miR-96-transfected cells could be rescued by overexpressing FOXO3a, while it was slightly restored by transfection with FOXO3a-3′-UTR (Figure 6B). Moreover, the MTT assay showed that co-transfection of miR-96 and FOXO3a significantly slowed the growth rate of both breast cancer cell lines (Figure 6C). However, the combination of FOXO3a-3′-UTR and miR-96 in the breast cancer cells had no obvious effect on the growth rate over that of breast cancer cells transfected only with miR-96 (Figure 6C). Taken together, our results suggest that suppression of FOXO3a plays an important role in promotion of cellular proliferation by miR-96.

To further examine whether the conclusions above could be supported by observations in human primary tumors, the expression levels of miR-96, p27^Kip1^, cyclin D1, and FOXO3a were examined in new freshly prepared two normal human breast tissues and eight human breast tumor tissues. As shown in Figure 6D and 6E, miR-96 expression and cyclin D1 mRNA expression were markedly upregulated, but the expression levels of p27^Kip1^ mRNA and FOXO3a protein were downregulated in breast tumors tissues as compared with those in normal human breast tissues. The miR-96 expression positively correlated with cyclin D1 (*r = *0.7392, *P<*0.01), whereas it negatively correlated with p27^Kip1^ (*r = *–0.7327, *P<*0.01). Furthermore, statistical analysis demonstrated that miR-96 expression not only inversely correlated with FOXO3a (r = −0.7577, *P*<0.001) but also significantly correlated with the Ki-67 level (r = 0.7151, *P*<0.001) (Figure 6E), which further strengthened the notion that miR-96 mediates proliferation of breast cancer through suppression of the transcriptional factor FOXO3a.

## Discussion

The key finding of the current study is that miR-96 expression was markedly upregulated in breast cancer cells and breast cancer tissues as compared with that in normal breast epithelial cells and normal breast tissues. We further found that ectopic expression of miR-96 could enhance the proliferation and anchorage-independent growth of breast cancer cells, while inhibition of miR-96 reduced these effects. Moreover, we demonstrated that upregulation of miR-96 in breast cancer cells led to the downregulation of cyclin-dependent kinase (CDK) inhibitors, p27^Kip1^ and p21^Cip1^, and upregulation of the cell-cycle regulator cyclin D1 through downregulation of FOXO3a via directly targeting the 3′-UTR of FOXO3a. These findings suggest that deregulation of miR-96 may play an important role in promoting carcinogenesis and progression of breast cancer.

It has been reported that more than one million women are diagnosed with breast cancer, and more than 400,000 patients die due to the disease in the world each year [30]. To date, breast cancer is the most common malignancy diagnosed among women worldwide, and it is the second leading cause of cancer death. Although both genetic and environmental factors are considered to be major causes of breast cancer, the molecular mechanism of its development and progression remain largely unknown [31]–[32]. As a member of the forkhead transcriptional factor family, FOXO3a is a key tumor suppressor in breast cancer [33]. It has been reported that upregulation of FOXO3a in breast cancer cells exerts a general inhibitory effect on cell growth [14]. Zou and colleagues demonstrated that functional interaction between FOXO3a and ER plays an important role in inhibiting estrogen-dependent breast cancer cell growth and tumorigenesis *in vivo* [34]. Activation of FOXO3a, by paclitaxel, antibodies against EGFR (cetuximab)/HER2 (trastuzumab), or mitogen-activated protein/extracellular signal-regulated kinase kinase (MEK) 1/2 inhibitor, results in apoptosis in breast cancer cells, indicating that activation of FOXO3a inhibits proliferation and development of breast cancer [35]–[37]. Consistent with these reports, downregulation of FOXO3a could significantly augment IKKβ-mediated tumor growth in nude mice and lead to apoptosis resistance in conjunction with elevated glycolysis that is the important for tumor growth and survival, suggesting that FOXO3a is a determinant of tumor progression and chemotherapeutic response [14], [38]. Collectively, all these studies indicate that activation of FOXO3a may be a potential therapeutic intervention strategy for breast cancer.

miRNAs, a class of small regulatory RNA molecules that negatively regulate their mRNA targets in a sequence-specific manner, have been demonstrated to play important roles in multiple biological processes, such as cellular differentiation, proliferation, oncogenesis, angiogenesis, invasion and metastasis, and can function as either tumor suppressors or oncogenes [22]–[24]. In the current study, we found that miR-96 was significantly overexpressed in breast cancer cell lines as compared with that in NBEC. Meanwhile, the expression of miR-96 was shown to be upregulated in breast cancer tissues in comparison to that in adjacent non-cancerous tissues from the same patient. Furthermore, we demonstrated that ectopic expression of miR-96 drastically increased the growth rate of MCF-7 and ZR-75-30 cells over that of control cells, while suppression of miR-96 inhibited cell proliferation and the colony-forming ability of the cells on soft agar, indicating that upregulation of miR-96 may correlate with clinical breast cancer progression and function as an onco-miRNA. In fact, the expression level of miR-96 is frequently upregulated in various human tumor types, including colorectal cancer, hepatocellular tumors (HCC) and chronic myeloid leukemia cells [39]–[41].

Through bioinformatics analysis, the tumor suppressor FOXO3a gene was indicated as a theoretical targeted gene of miR-96. We were able to determine that FOXO3a is a *bona fide* target of miR-96 by three different methods. Western blotting analysis showed that overexpression of miR-96 resulted in the decrease of FOXO3a protein. Real-time PCR analysis determined that the downstream targets of FOXO3a, including cell-cycle inhibitors p21^Cip1^ and p27^Kip1^ were significantly downregulated, while the regulator cyclin D1 was upregulated in miR-96 transfected breast cancer cells. The luciferase activity assay and point mutation analysis demonstrated that the downregulation of FOXO3a was mediated by miR-96 through the FOXO3a-3′UTR. Furthermore, ectopically expressing FOXO3a (without 3′UTR) significantly abrogated the miR-96-induced proliferation, but transfection with FOXO3a-3′-UTR (with 3′UTR) only partially attenuated the proliferative enhancement by miR-96 overexpression, suggesting that the effect of miR-96 on proliferation of breast cancer cells may be though downregulation of FOXO3a via directly targeting the FOXO3a 3′-UTR. The biological function of miR-96 in protection against apoptosis and cell survival, as related to FOXO3a function, is currently under investigation in our laboratory.

Recently, several miRs were found to be involved in the regulation of the FOXO family of transcription factors. It was reported that miR-27a, miR-96, and miR-182 coordinately regulate the expression of FOXO1 through directly targeting the FOXO1 3′-UTR [42]. miR-155 was shown to regulate cell survival, growth and hemosensitivity by downregulating FOXO3a in breast cancer [27]. Wang and colleagues reported that upregulation of FOXO3a results in a decrease in miR-21 expression and suppressing its oncogenic activity, which further supports the notion that FOXO3a functions as a tumor suppressor [43]. Interestingly, Yu and colleagues found that miR-96 was downregulated in pancreatic cancer and functions as a tumor suppressor gene. However, we did not observe the obvious change of KRAS expression in breast cancer cells transfected with miR-96 (Data not shown), suggesting that a single miRNA may have several distinct functions in different cell types, which likely depends on the availability of specific targets or downstream effectors, as suggested by Hyun *et al*. and Betel *et al.* [44]–[45]. Indeed, it has been reported that miR-26 is amplified in high-grade glioma and facilitates gliomagenesis through downreregulation of the tumor suppressor PTEN [46]. However, Ji and colleagues found that miR-26 was downregulated and was associated with hepatocellular carcinoma via activation of the NF-κB pathway [47]. Meanwhile, overexpressing miR-30 could inhibit apoptosis through repression of p53 expression in cardiomyocytes [48], but upregulation of miR-30 in breast cancer cells induced apoptosis by targeting Ubc9 [49].

In summary, the current study provides, for the first time, an important link between miR-96-mediated proliferation of breast cancer cells and downregulation of FOXO3a. Our findings suggest an essential role of miR-96 in the regulation of breast cancer cells proliferation. Understanding the precise role played by miR-96 in breast cancer progression will not only increase our knowledge of the biology of the tumor but its inhibition may also allow development of a novel therapeutic strategy.

## Supporting Information

Figure S1 **FOXO3a is downregulated in breast cancer cells.** Western blotting analysis of FOXO3a expression in normal breast epithelial cells (NBECs) and breast cancer cell lines, including BT549, ZR-75-30, Bcap37, MDA-MB231, MDA-MB435, MCF-7, SKBR3, MDA-MB453 and T47D. α-tubulin was used as a loading control.
(TIF)
