# Supplementary figures and images for: Unregulated miR-96 Induces Cell Proliferation in Human Breast Cancer by Downregulating Transcriptional Factor FOXO3a
Source: PLoS One. 2010 Dec 23;5(12):e15797. doi: 10.1371/journal.pone.0015797 (PMC3009749; doi:10.1371/journal.pone.0015797)

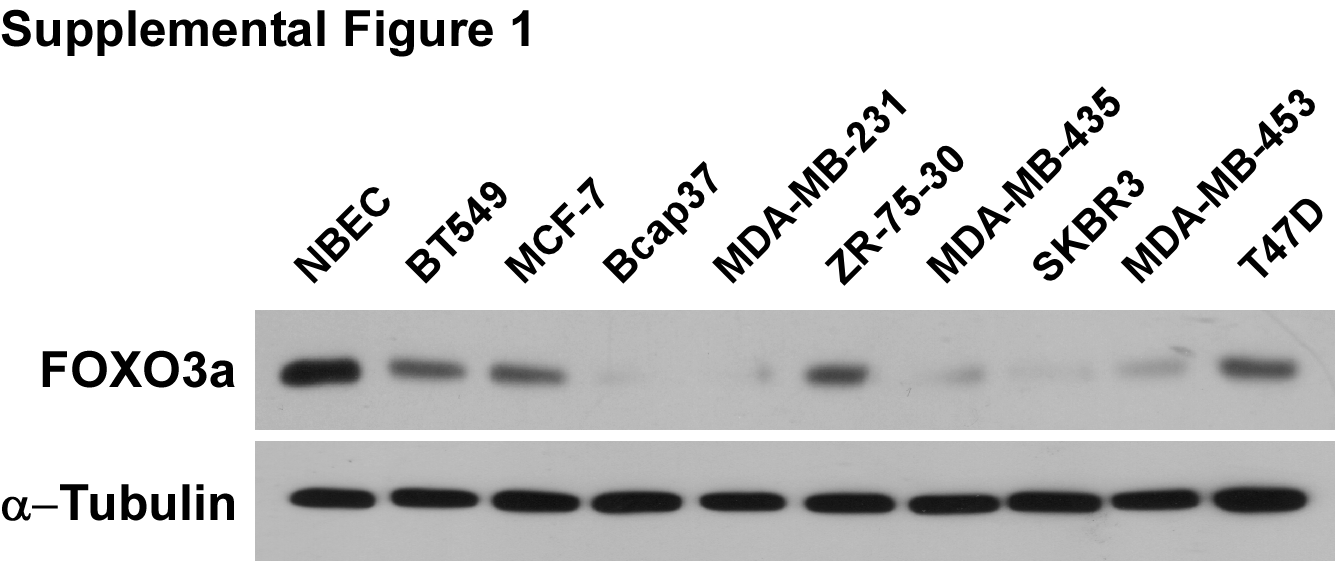

Supplement: Figure S1 — FOXO3a is downregulated in breast cancer cells. Western blotting analysis of FOXO3a expression in normal breast epithelial cells (NBECs) and breast cancer cell lines, including BT549, ZR-75-30, Bcap37, MDA-MB231, MDA-MB435, MCF-7, SKBR3, MDA-MB453 and T47D. α-tubulin was used as a loading control. (TIF) [file pone.0015797.s001.tif]
